# Efficacy and Safety of Multidisciplinary Sandwich Regimen in AQP4‐IgG+ NMOSD With Preconception B‐Cell Depletion During Perigravidity

**DOI:** 10.1002/cns.71222

**Published:** 2026-10-23

**Authors:** Li Xiao, Gengxin Lu, Qian Gao, Feiyan Wan, Zhenze Lu, Wei Qiu, Chengfang Xu, Yaxin Lu, Lejia Xu, Tingting Xia, Jiajing Chao, Yu Fu, Zhuanggui Chen, Rui Li, Yanyu Chang, Yuge Wang, Bing Qin, Yaqing Shu

**Affiliations:** ^1^ Department of Neurology The Third Affiliated Hospital of Sun Yat‐Sen University Guangzhou China; ^2^ Department of Obstetrics The Third Affiliated Hospital of Sun Yat‐Sen University Guangzhou China; ^3^ Department of Neurology Guangzhou Eighth People's Hospital, Guangzhou Medical University Guangzhou China; ^4^ Department of Neurology, Neuromedicine Center the University of Hong Kong‐Shenzhen Hospital Shenzhen China; ^5^ Big Data and Artificial Intelligence Center The Third Affiliated Hospital of Sun Yat‐Sen University Guangzhou China; ^6^ Department of Pharmacy The Third Affiliated Hospital of Sun Yat‐Sen University Guangzhou China; ^7^ Center for Reproductive Medicine The Third Affiliated Hospital of Sun Yat‐Sen University Guangzhou China; ^8^ Children Medical Center The Third Affiliated Hospital of Sun Yat‐Sen University Guangzhou China

**Keywords:** B‐cell depleting agents, inebilizumab, neuromyelitis optica spectrum disorder, pregnancy, rituximab, sandwich regimen

## Abstract

**Background:**

Neuromyelitis optica spectrum disorder (NMOSD) predominantly affects women of childbearing age, accompanied by markedly elevated relapse risks during pregnancy and postpartum periods, bringing great challenges to perigravid management. At present, there is no standardized gestational therapeutic regimen for NMOSD worldwide, and data on perigravid application of B‐cell depleting agents including rituximab and inebilizumab remain limited. For patients exposed to B‐cell depleting agents before conception, balancing disease control and fetal safety is an urgent clinical problem to be solved.

**Objective:**

This multicenter prospective cohort study aimed to evaluate the efficacy and safety of a three‐stage multidisciplinary “sandwich” management regimen in AQP4‐IgG‐positive pregnant women with NMOSD exposed to B‐cell depleting agents before conception. Maternal disease activity, pregnancy outcomes, neonatal conditions, and offspring growth and developmental characteristics within the first year of life were analyzed.

**Methods:**

A total of 8 AQP4‐IgG‐positive pregnant women with NMOSD who received B‐cell depleting agents within 9 months prior to conception were enrolled from multiple centers between January 2022 and October 2024, including 3 patients treated with inebilizumab and 5 patients treated with rituximab. All subjects received a unified three‐stage intervention: B‐cell depleting agents were discontinued upon confirmed pregnancy; low‐dose azathioprine combined with low‐dose glucocorticoids was administered during gestation; B‐cell depleting agents were reinitiated at 2 weeks postpartum. Multidisciplinary teams consisting of neurology, obstetrics, clinical pharmacy, and neonatology departments conducted full‐cycle monitoring and follow‐up. Primary observation indicators included maternal relapse events, Expanded Disability Status Scale (EDSS) scores, B‐cell counts, gestational complications, gestational age at delivery, neonatal physical indicators, Apgar scores, congenital malformations, as well as offspring growth and neurodevelopment and vaccination safety from birth to 1 year of age.

**Results:**

All subjects achieved term live births without severe obstetric complications. Seven patients remained relapse‐free throughout pregnancy and postpartum period with stable or improved EDSS scores; one patient discontinued all medications during gestation and suffered a relapse at 1 month postpartum. Neonatal physical indicators and Apgar scores were generally normal, one neonate presented mild hypospadias and two developed transient neonatal abnormalities; neonatal B‐cell counts at birth and 1 month of age were within normal ranges. One‐year follow‐up showed normal physical and neurodevelopment in all offspring with safe vaccination procedures.

**Conclusions:**

For AQP4‐IgG‐positive pregnant women with NMOSD exposed to B‐cell depleting agents before conception, the multidisciplinary “sandwich” regimen featuring B‐cell depletion before conception and postpartum plus gestational bridging therapy with low‐dose azathioprine combined with glucocorticoids is safe and effective, reduces disease relapse risk and yields favorable overall maternal and fetal outcomes. This regimen may serve as a promising perigravid management strategy that warrants further validation in larger cohorts.

## Graphical abstract

We described a multidisciplinary “sandwich” regimen combining preconception B‐cell depletion, gestational AZA‐steroid bridging and postpartum retreatment. In 7 of 8 AQP4‐IgG‐positive NMOSD women, this strategy achieved good maternal relapse control, together with favorable fetal safety and normal infant neurodevelopment within one‐year follow‐up.

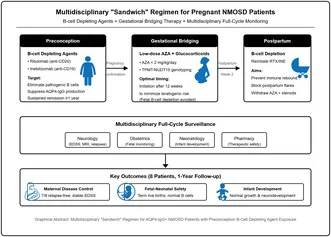

## 1 Introduction

Neuromyelitis optica spectrum disorder (NMOSD) is an autoimmune disease of the central nervous system mediated by AQP4‐IgG, characterized by high relapse and high disability rates [1, 2]. The disease mainly occurs in women aged 20–40 years, whose peak incidence overlaps heavily with the female reproductive cycle [3]. Disease relapse risk rises significantly during pregnancy and postpartum periods [4, 5, 6]. Disease activity during the perigravid period may cause irreversible permanent neurological deficits in mothers, greatly increase the risks of adverse pregnancy events such as miscarriage and preterm delivery, and threaten both maternal and fetal health [7]. Difficulties in perigravid intervention have become an urgent clinical bottleneck at the intersection of neuroimmunology and obstetrics.

Globally, no unified standardized perigravid management protocol for NMOSD has been established to date. Existing therapeutic recommendations are mostly based on small retrospective cohorts and expert consensus with insufficient high‐level evidence [8, 9, 10, 11]. Meanwhile, traditional immunosuppressants such as mycophenolate mofetil and methotrexate carry definite teratogenic risks, leaving limited therapeutic options for clinical use during gestation [8, 12, 13, 14].

B‐cell depleting agents represented by rituximab and inebilizumab can target and eliminate pathogenic B cells and inhibit AQP4‐IgG production, which have become first‐line core therapies for AQP4‐IgG‐positive NMOSD with expanding clinical application [8, 9, 11, 15, 16, 17]. Drug labels generally recommend a 6–12‐month washout period before conception [18, 19], yet real‐world perigravid data of these agents remain scarce, especially prospective gestational data of inebilizumab, a novel anti‐CD19 monoclonal antibody [15]. With the widespread clinical use of B‐cell depleting agents, an increasing number of patients become pregnant after preconception exposure [16, 20], while standardized intervention strategies balancing stable disease control and fetal safety are lacking in clinical practice.

To address the above clinical dilemmas and critical gaps in evidence, we conducted this multicenter prospective cohort study, consecutively enrolling AQP4‐IgG‐positive pregnant women with NMOSD exposed to B‐cell depleting agents within 9 months before conception. We innovatively proposed a three‐stage “sandwich” intervention model covering preconception, gestational and postpartum periods, and built multidisciplinary teams of neurologists, obstetricians, clinical pharmacists, and neonatologists to carry out integrated full‐cycle follow‐up management. Stratified analysis based on the timing of gestational bridging therapy initiation was performed to systematically evaluate the efficacy and safety of this regimen. This study aims to provide evidence‐based references for perigravid management of NMOSD patients with prior B‐cell depleting agent exposure and facilitate the construction of standardized full‐cycle perigravid management systems for this special population.

## 2 Subjects and Methods

### 2.1 Study Design

This was a multicenter, prospective, observational cohort study that consecutively enrolled AQP4‐IgG‐positive pregnant women with NMOSD exposed to B‐cell depleting agents within 9 months before conception. All patients were recruited in the first trimester and followed up until 1 year postpartum.

This study was conducted in accordance with the principles of the Declaration of Helsinki, and the protocol was approved by the medical ethics committee at the Third Affiliated Hospital of Sun Yat‐Sen University (approval number: [2021]02‐400‐01) and the medical ethics committee of the University of Hong Kong‐Shenzhen Hospital (approval number: [2024]200).

All participants (or their legal guardians for minors) provided written informed consent to participate in this study.

### 2.2 Study Subjects

Pregnant women with NMOSD treated from January 2022 to October 2024 at the Third Affiliated Hospital of Sun Yat‐Sen University and the University of Hong Kong‐Shenzhen Hospital were enrolled. Inclusion criteria: Meeting the 2015 international diagnostic criteria for NMOSD with positive AQP4‐IgG; exposure to B‐cell depleting agents (rituximab or inebilizumab) within 9 months before conception; singleton pregnancy with complete follow‐up accessible from the first trimester to 1 year postpartum; complete baseline, gestational monitoring, obstetric and offspring follow‐up data. Exclusion criteria: Combined other autoimmune diseases; multiple gestation; incomplete key clinical data or loss to follow‐up.

### 2.3 Unified “Sandwich” Management Regimen

All participating centers implemented a standardized three‐stage prospective perigravid “sandwich” regimen. Subjects received B‐cell depleting agents within 9 months before conception, which were immediately discontinued upon confirmed pregnancy. Low‐dose azathioprine (daily dose < 2 mg/kg) combined with low‐dose glucocorticoids served as the core bridging therapy, which could be initiated before conception, within the first trimester or after 12 weeks of gestation. Medication decisions were made via standardized shared patient‐physician decision‐making: the multidisciplinary team (neurology, obstetrics, and clinical pharmacy) fully informed patients of the risks of disease relapse and the benefits and risks of immunosuppressive bridging therapy; patients' preferences and clinical conditions were comprehensively discussed, and the final therapeutic plan was documented in medical records after signed informed consent. Only one patient refused all immunosuppressive therapy during gestation and did not restart B‐cell depleting agents postpartum; this treatment choice was pre‐specified and permitted in the study protocol rather than a protocol deviation, as the protocol allowed patients to decline gestational immunosuppressive bridging therapy after full risk–benefit discussion and signed informed consent. All other subjects reinitiated B‐cell depleting agents at 2 weeks postpartum under full‐cycle multidisciplinary surveillance of neurology, obstetrics, clinical pharmacy, and neonatology teams. All patients scheduled for azathioprine therapy underwent high‐throughput sequencing of thiopurine metabolism genes *TPMT* and *NUDT15* to assess medication risks. Regular follow‐up visits were arranged during the first, second and third trimesters, delivery and postpartum periods to evaluate disease activity, monitor B‐cell counts and various obstetric indicators. Azathioprine and glucocorticoids were simultaneously withdrawn when B‐cell depleting agents were reintroduced at 2 weeks postpartum. Combined maternal‐offspring follow‐up assessments were conducted at 1, 3, 6, and 12 months postpartum.

### 2.4 Observation Indicators

Observation indicators were divided into three categories:

Maternal indicators: age at conception, disease duration, number of preconception relapses, perigravid therapeutic regimens, preconception Expanded Disability Status Scale (EDSS) scores, serial hematologic, hepatic, renal, and serum AQP4‐IgG laboratory parameters (preconception, gestational, and postpartum), gestational and postpartum disease relapses, changes in EDSS scores throughout perigravid period, gestational complications, delivery mode, anesthesia modality, and paired preconception and postpartum brain, optic nerve, and spinal cord magnetic resonance imaging (MRI).

Serum AQP4‐IgG was measured by a cell‐based assay (CBA), as described in our previous study [21].

Pregnancy and neonatal indicators: gestational age at delivery, neonatal birth weight, body length, 1‐ and 5‐min Apgar scores, congenital malformations, neonatal complications, and feeding patterns.

Long‐term offspring evaluation indicators: routine blood test results at birth and 1 month of age, offspring physical growth from birth to 1 year, Michaelis early childhood developmental scores, Ages & Stages Questionnaires, 3rd edition (ASQ‐3) assessment results, infection incidence, antibiotic usage and vaccination safety.

### 2.5 Statistical Methods

Medians were reported for measurement data, and cases (percentages) for enumeration data. Descriptive statistical analyses were performed using SPSS 26.0 software.

## 3 Results

### 3.1 Baseline Characteristics of Patients

Eight AQP4‐IgG‐positive pregnant women with NMOSD were enrolled, with a median conception age of 30.5 years, median disease duration of 6.23 years and median 4.5 preconception relapses. All patients received B‐cell depleting agents before conception, including 3 patients treated with 300 mg inebilizumab and 5 patients treated with 300–500 mg rituximab. The median interval from the last infusion of B‐cell depleting agents to the last menstrual period (LMP) was 3.74 months, confirming all infusions were administered within 9 months before conception. The median cumulative duration of B‐cell depleting therapy before conception was 7.22 months. All patients except Patient 6 and Patient 7 achieved B‐cell depletion status at conception (defined as CD19^+^ B cell percentage < 1%) (Table 1, Figure 1). Stratification by gestational medication: Patients 5 and 8 initiated azathioprine combined with glucocorticoids before conception; Patients 1, 4, and 7 initiated therapy within the first trimester; Patients 2 and 6 initiated therapy after 12 weeks of gestation; Patient 3 received no immunosuppressants during gestation (Table 1, Figure 2). Genetic testing of *TPMT* and *NUDT15* showed normal enzymatic activity in all subjects with low myelosuppression risk, supporting standard‐dose azathioprine treatment. Complete blood counts, liver and renal function tests remained normal for all mothers throughout preconception, gestational and postpartum periods (Table S1).

**TABLE 1:** Maternal characteristics of the study sample.

Mat	1^a^	2	3	4	5	6	7	8	Median
Age at conception (years)	27	29	36	39	33	24	27	32	30.5
Ht at conception (cm)	156	148	157	155	163	161	165	152	156.5
Wt at conception (kg)	55	49	48	42	52	60	78.5	65	53.5
Body surface area (m^2^)	1.73	1.42	1.45	1.35	1.53	1.64	1.90	1.66	1.59
GW	38 + 5	39	40 + 1	38 + 2	38 + 2	38 + 6	38 + 5	38 + 4	
Disease duration at conception in years	4.15	6.07	8.2	5.9	14.78	2.55	9.14	6.39	6.23
Relapse episodes before conception	5	4	4	4	10	4	7	5	4.5
Duration of remission before conception (years)	0.82	0.65	0.48	1.07	1.28	0.93	1.88	1.3	1
Drug exposure	Inebilizumab 300 mg	Inebilizumab 300 mg	Inebilizumab 300 mg	RTX 300 mg	RTX 500 mg	RTX 500 mg	RTX 500 mg	RTX 500 mg	—
Pre‐pregnancy EDSS score	3	3	2	0	3.5	1	4	2	2.5
Interval between last B‐cell depleting agent and LMP (months)	−0.63	−1.73	−5.1	−5.57	−3.37	−1.67	−4.1	−7.9	−3.74
Cumulative B‐cell depletion therapy duration (months)	6.96	6.13	0.7	7.47	12.2	9.63	18.03	0.9	7.22
Medication in pregnancy	MP 4 mg qd + AZA 50 mg bid	MP 8 mg qd + AZA 50 mg bid	None	MP 8 mg qd + AZA 40 mg bid	PDN 10 mg qd + AZA 50 mg bid	MP 8 mg qd + AZA 50 mg bid	MP 8 mg qd + AZA 50 mg bid	MP 8 mg qd + AZA 50 mg bid	—
Interval from GC & AZA use to LMP (months)	1.7	5.33	—	1.47	−3.07	3.73	1.8	−2.43	1.7
Delivery mode	Cesarean section	Cesarean section	Vaginal delivery	Cesarean section	Cesarean section	Vaginal delivery	Cesarean section	Cesarean section	—
Pregnancy complications	Grade III MSAF	GDM	GDM	No	No	Polyhydramnios	GBS carrier	GBS carrier & VVC	—
Pregnancy and postpartum clinical relapses	No	No	Yes	No	No	No	No	No	
Postpartum EDSS score	3	2	1	0	3	1	3	2	2

Abbreviations: AZA, azathioprine; CSEA, combined spinal‐epidural anesthesia; GBS, group B streptococcal; GC, glucocorticoid; GDM, gestational diabetes mellitus; GW, gestational week at birth; Ht., height; Mat., maternal; MP, methylprednisolone; MSAF, meconium‐stained amniotic fluid; PDN, prednisone; VVC, vulvovaginal candidiasis; Wt., weight.

^a^ The clinical data of Patient 1 has been previously reported as an independent case report published in BMC Neurology.

**FIGURE 1:**
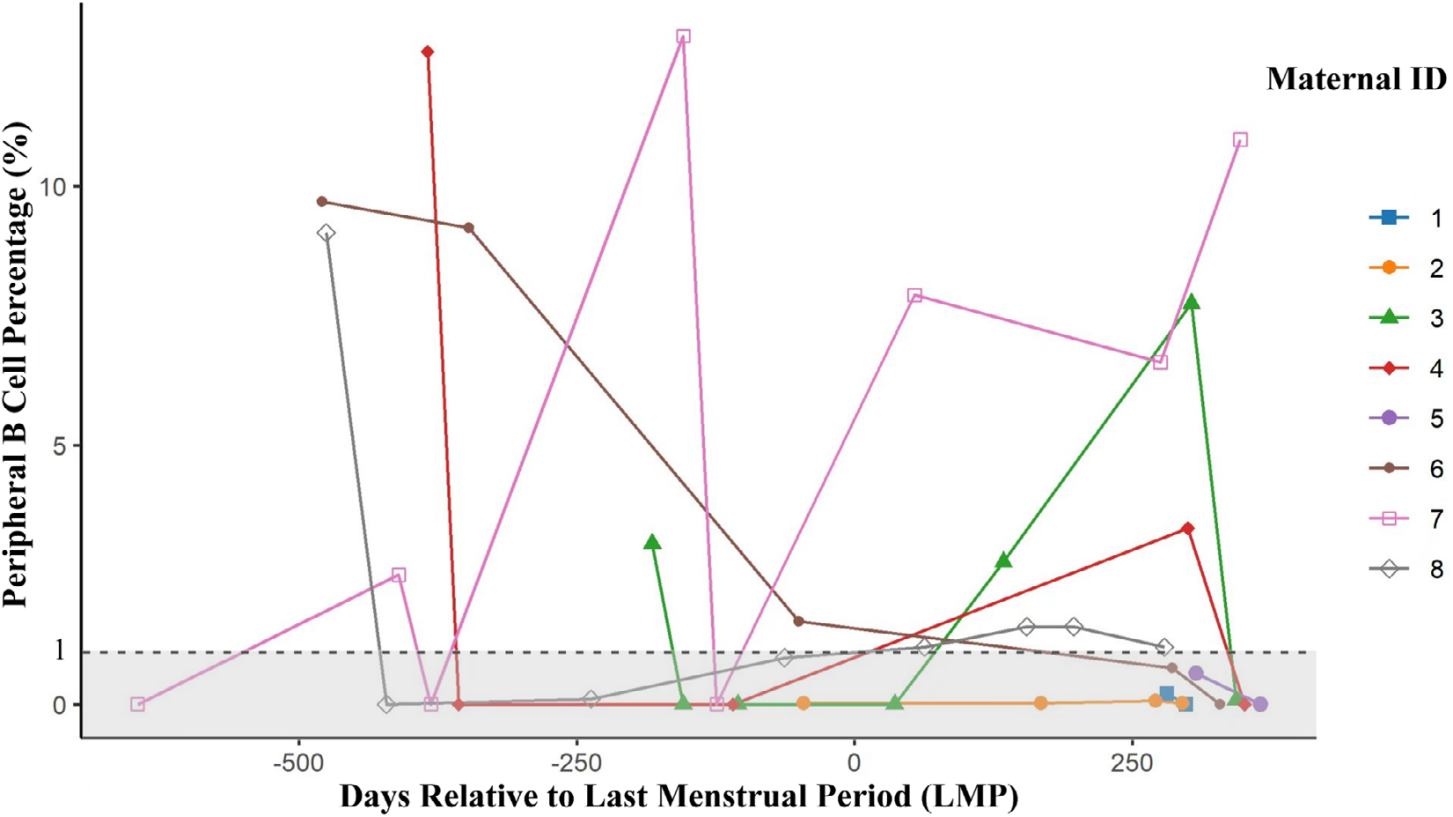
Dynamic changes in peripheral B‐cell percentage in 8 pregnant women with AQP4‐IgG‐positive NMOSD, from preconception through pregnancy and postpartum follow‐up. The *x*‐axis shows days relative to the last menstrual period (LMP); the *y*‐axis shows B‐cell percentage (%). The dashed line marks the B‐cell depletion threshold (< 1%). Each curve represents one patient's trajectory. All patients achieved B‐cell depletion at conception, with individual variation in B‐cell recovery during pregnancy and postpartum.

**FIGURE 2:**
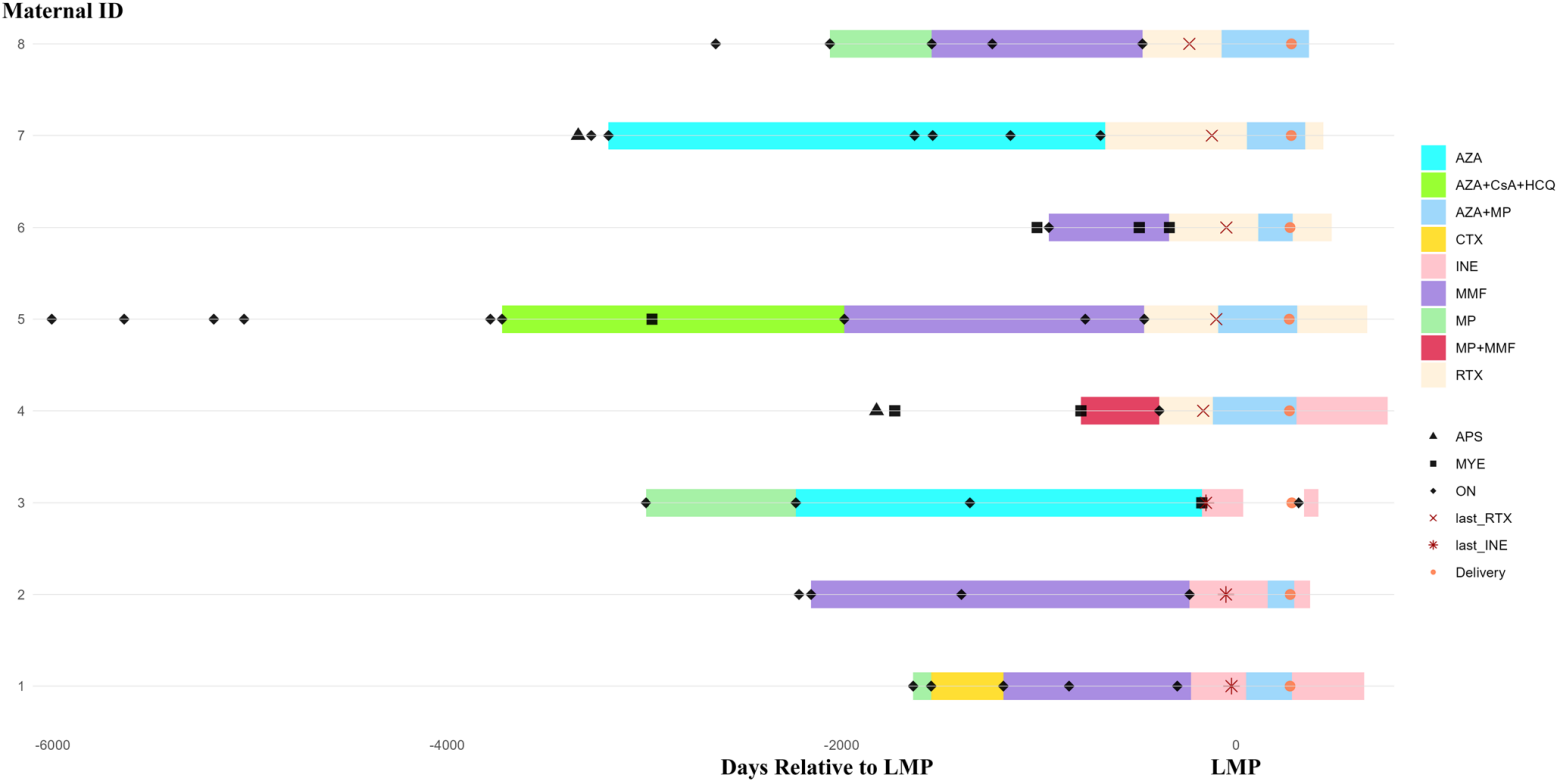
Treatment regimen and relapse trajectory in periconceptional women with NMOSD. The horizontal axis represents days relative to the last menstrual period (LMP), with LMP set as time zero and negative values indicating the preconception interval; the vertical axis denotes unique maternal identification numbers (1–8). Colored horizontal bars indicate sequential therapeutic regimens administered throughout the clinical course: Azathioprine (AZA), AZA combined with cyclosporine A and hydroxychloroquine (AZA + CsA + HCQ), AZA combined with methylprednisolone (AZA + MP), cyclophosphamide (CTX), inebilizumab (INE), mycophenolate mofetil (MMF), methylprednisolone (MP), MP combined with MMF (MP + MMF), and rituximab (RTX). Distinct symbols mark key clinical and treatment timepoints: Triangle (▲) = acute disseminated encephalomyelitis‐like attack (APS), solid square (■) = myelitis (MYE), diamond (◆) = optic neuritis (ON), cross (×) = date of last rituximab infusion (last_RTX), asterisk (*) = date of last inebilizumab infusion (last_INE), solid orange circle (●) = delivery date.

### 3.2 Maternal Disease Activity and EDSS Changes

The median duration of sustained remission before conception was 1 year. Patient 3 discontinued all medications during gestation and failed to restart disease‐modifying therapy (DMT) postpartum, presenting a clinical relapse at 1 month postpartum that remitted after glucocorticoid intervention. The remaining seven patients experienced no clinical relapses throughout pregnancy and the first postpartum year, with stable or improved EDSS scores and no disability progression (Table 1, Figure 3). No interval changes in central nervous system demyelinating lesions were detected between preconception and postpartum MRI scans in all patients (Figure S2).

**FIGURE 3:**
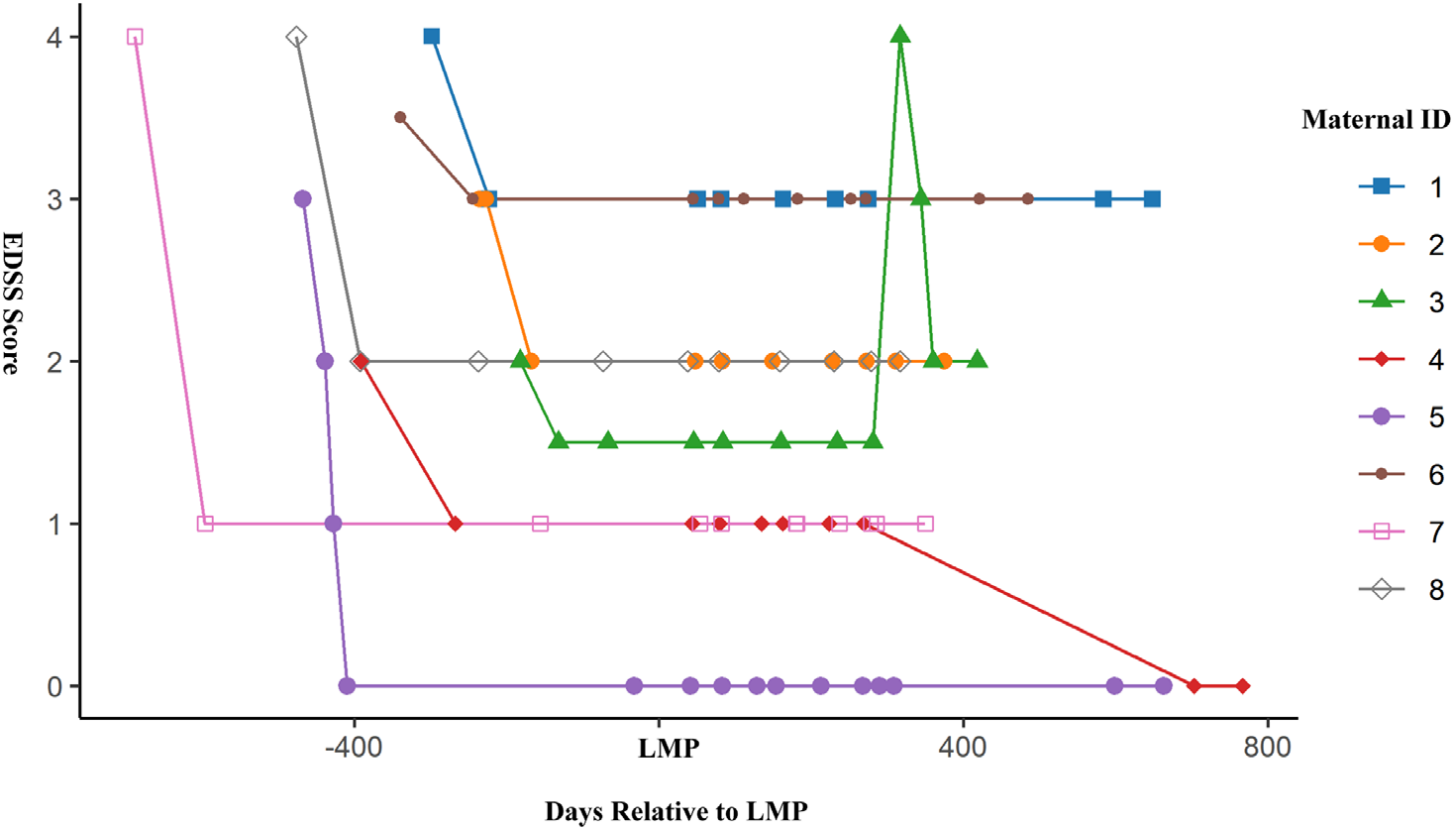
EDSS score trajectories of 8 women with AQP4‐IgG‐positive NMOSD from preconception through pregnancy and postpartum. The *x*‐axis shows days relative to the last menstrual period (LMP), and the *y*‐axis shows EDSS scores. Each curve represents one patient. Most patients maintained stable or improved disability status, with only one transient postpartum EDSS increase and no permanent progression.

### 3.3 Gestational Complications and Delivery Outcomes

All subjects delivered viable term neonates at a median gestational age of 38 + 5 weeks. No severe adverse obstetric events including severe preeclampsia, placental abruption, severe postpartum hemorrhage or stillbirth were observed. Minor gestational complications included grade III meconium‐stained amniotic fluid, gestational diabetes mellitus, polyhydramnios, group B streptococcus colonization and vulvovaginal candidiasis. Six patients received cesarean delivery and two delivered vaginally (Table 1).

### 3.4 Neonatal Birth Outcomes

Median neonatal birth weight and body length were 3105 g and 50 cm respectively. Only neonates of Patient 1 and Patient 8 presented slightly decreased Apgar scores; the other six neonates obtained full 10‐point scores for both 1‐ and 5‐min Apgar assessments (Table 2). The neonate of Patient 1, who initiated azathioprine‐glucocorticoid therapy within the first trimester, developed mild hypospadias, hyperbilirubinemia, and mild anemia. The neonate of Patient 8 was diagnosed with neonatal pneumonia and received antibiotic treatment. All other newborns showed unremarkable clinical manifestations (Table 2).

**TABLE 2:** Pregnancy and offspring outcomes.

Child	1	2	3	4	5	6	7	8	Median
Pregnancy and neonatal outcomes									
GW at birth	38 + 5	39	40 + 1	38 + 2	38 + 2	38 + 6	38 + 5	38 + 4	38 + 5
Gender	M	M	F	F	M	M	F	F	—
Birth weight (g)	2800	3010	3750	3650	3200	3650	3000	2750	3105
Birth length (cm)	48	50	50	52	51	52	49	48	50
Major congenital abnormality	Hypospadias	No	No	No	No	No	No	No	—
1‐min Apgar score	8	10	10	10	10	10	10	7	10
5‐min Apgar score	8	10	10	10	10	10	10	10	10
Prolonged hospital stay immediately after birth	Yes	No	No	No	No	No	No	Yes	—
Neonatal diseases	Hyperbilirubinemia, Mild anemia	None	None	None	None	None	None	Neonatal pneumonia	—
Systemic antibiotic use	No	No	No	No	No	No	No	Yes	—
Infant outcomes									
Feeding mode	Artificial feeding	Artificial feeding	Artificial feeding	Artificial feeding	Artificial feeding	Artificial feeding	Artificial feeding	Breast feeding	—
Developmental delay	No	No	No	No	No	No	No	No	—
Reported developmental delay	No	No	No	No	No	No	No	No	—
Conspicuous early child development score (Michaelis)	No	No	No	No	No	No	No	No	—
Systemic antibiotic use during follow‐up	Pneumonia at 4.5 months, treated with antibiotics, without hospitalization	No	No	Fever at 6 months, no antibiotics used	No	No	No	No	—
Vaccination status^a^	On schedule; no adverse reactions	On schedule; no adverse reactions	On schedule; no adverse reactions	On schedule; no adverse reactions	On schedule; no adverse reactions	On schedule; no adverse reactions	On schedule; no adverse reactions	On schedule; no adverse reactions	—

Abbreviations: F, female; GW, gestational week; M, male.

^a^ All infants received the following live vaccines within one year of age: Bacillus Calmette‐Guérin vaccine (BCG), rotavirus vaccine (RV), measles‐rubella vaccine (MR), live attenuated Japanese encephalitis vaccine (JE‐L), measles‐mumps‐rubella vaccine (MMR), and live attenuated varicella vaccine (VAR).

### 3.5 Neonatal and Infant B‐Cell Status

Complete blood counts at birth were within normal reference ranges for all neonates: median white blood cell count 15.73 × 10^9^/L, median hemoglobin 166.5 g/dL, median platelet count 287.5 × 10^9^/L; median B‐cell percentage was 17.65%, falling within the normal reference interval. At the 1‐month follow‐up, complete blood counts and B‐cell levels remained normal in all infants without persistent B‐cell reduction or hematological abnormalities (Table S2).

### 3.6 Offspring Growth, Development and Safety Within the First Year of Life

At the 1‐year follow‐up, weight, body length, and head circumference of all offspring fell within age‐matched normal ranges (Figure S1). Michaelis early developmental scores and ASQ‐3 assessments revealed no developmental abnormalities. All offspring completed routine national immunization schedules including BCG, RV, MR, JE‐L, MMR, and VAR live‐attenuated vaccines without severe post‐vaccination adverse reactions. Only the infant of Patient 1 developed pneumonia at 4.5 months of age and recovered fully after antibiotic therapy. The infant of Patient 4 experienced self‐limited transient fever at 6 months without antibiotic administration. No severe infectious episodes were recorded in the other six offspring during follow‐up (Table 2).

## 4 Discussion

This multicenter prospective cohort study is the first worldwide to focus on AQP4‐IgG‐positive pregnant women with NMOSD exposed to B‐cell depleting agents before conception. We proposed an innovative three‐stage “sandwich” perigravid management regimen, whose core protocol consists of B‐cell depletion before conception and postpartum, with low‐dose azathioprine (AZA) combined with glucocorticoids as gestational bridging therapy. Multidisciplinary full‐cycle management enables comprehensive risk control for mothers and offspring. A total of 8 eligible patients were enrolled in this study, and follow‐up results verified the overall safety and feasibility of this regimen, providing high‐quality real‐world evidence for standardized perigravid management of this special population. In addition, three patients received inebilizumab in our cohort, supplementing scarce global prospective gestational safety data of this agent and enriching clinical evidence resources.

The “sandwich” regimen fully integrates the immunopathogenesis of NMOSD and gestational physiological changes, drawing on successful experience of anti‐CD20 therapy perigravid management in multiple sclerosis (MS) [22, 23, 24]. B‐cell depleting agents can profoundly eliminate B lymphocytes and suppress the production of pathogenic AQP4‐IgG, serving as core medications for preventing NMOSD relapses [2]. Although fetal exposure to B‐cell depleting agents exists theoretically, placental transport capacity is limited in the first trimester, which restricts drug transfer into fetuses and avoids disruption of fetal immune development. However, increased placental barrier permeability during the second and third trimesters allows transplacental drug delivery and may cause fetal B‐cell depletion, so continuous administration throughout gestation is not feasible [25, 26]. Accordingly, our regimen discontinues B‐cell depleting agents upon confirmed pregnancy and leverages their long‐lasting immunosuppressive effects. Four patients in our cohort maintained sustained B‐cell depletion throughout gestation, which effectively covered the teratogenic high‐risk first trimester.

Low‐dose azathioprine combined with glucocorticoids was selected as gestational bridging therapy, with sufficient gestational safety evidence, low placental transfer rates and controllable teratogenic risks [20, 27, 28, 29, 30]. Azathioprine smoothly fills the immunosuppressive gap after discontinuation of B‐cell depleting agents to prevent immune rebound and disease flares, while glucocorticoids synergistically inhibit mild inflammatory fluctuations [17, 31]. The combination of the two drugs complements each other to sustain stable disease control during gestation. Seven patients receiving gestational bridging therapy remained relapse‐free throughout pregnancy and the first postpartum year with stable or improved EDSS scores and no disability progression, fully verifying the long‐term maternal disease control efficacy of this regimen.

Stratified analysis based on the timing of gestational AZA‐glucocorticoid initiation showed comparable disease control efficacy across preconception, first‐trimester and post‐12‐week initiation groups, with divergent safety profiles. Although based on a small sample, these findings suggest that initiating therapy after 12 weeks of gestation may be associated with sustained disease control and no apparent adverse neonatal outcomes, supporting a potentially favorable safety profile for this timing. While disease control remained satisfactory in patients initiating therapy before conception or within the first trimester, one neonate developed mild hypospadias, highlighting the necessity of enhanced prenatal screening for this subgroup, while current evidence cannot confirm a direct causal relationship between this malformation and the medication regimen. Collectively, bridging therapy initiated after 12 weeks of gestation better avoids teratogenic risks during the critical embryonic period; this exploratory finding requires validation in larger prospective cohorts. Patients already established on this regimen before conception may continue treatment under close maternal‐fetal surveillance. Furthermore, complete withdrawal of immunosuppression during gestation significantly elevates relapse risk, requiring individualized therapeutic regimens in clinical practice.

All enrolled patients delivered viable term neonates. Minor gestational complications including meconium‐stained amniotic fluid, gestational diabetes mellitus, and group B streptococcus colonization are common obstetric complications with natural background incidence in general pregnant populations. All neonates presented normal physical development, Apgar scores, and peripheral B‐cell counts. One‐year longitudinal follow‐up of offspring showed unimpaired physical and neurodevelopment, with successful administration of all live‐attenuated vaccines and no severe adverse reactions, indicating acceptable short‐term fetal safety under this management regimen. Of note, preconception maternal B‐cell depletion may theoretically alter offspring immune competence [32]; our cohort provides preliminary real‐world safety observations for live attenuated vaccination in this high‐risk offspring population.

Combining our cohort data with published perigravid management evidence of multiple sclerosis [22, 33], AQP4‐IgG‐positive women of childbearing age with frequent relapses, prior disabling attacks and persistently high AQP4‐IgG titers face markedly elevated relapse risks upon complete discontinuation of disease‐modifying therapy during gestation [34]. We recommend implementing the full perigravid “sandwich” regimen for this high‐risk population: sustained B‐cell depleting therapy for at least 1 year before conception to stabilize disease activity, discontinuation upon confirmed pregnancy, bridging therapy with low‐dose azathioprine plus glucocorticoids initiated after 12 weeks of gestation, and timely reintroduction of B‐cell depleting agents postpartum. This strategy relies on sustained preconception B‐cell depletion to provide long‐term immune protection during the teratogenic high‐risk first trimester, uses gestational bridging therapy to fill immunosuppressive gaps and block postpartum immune rebound, and avoids fetal exposure to B‐cell depleting agents in late gestation. The regimen minimizes perigravid disease activity risks while safeguarding maternal and fetal safety, which may serve as a preferred intervention strategy for high‐risk reproductive‐aged patients with NMOSD.

Full‐cycle monitoring and management were carried out by multidisciplinary teams consisting of neurology, obstetrics, neonatology, and clinical pharmacy departments throughout the study, which also reflects the critical role of interdisciplinary collaboration in perigravid diagnosis and treatment of NMOSD.

Due to the rarity of NMOSD, the sample size of pregnant patients with NMOSD enrolled in this study was limited. The small sample precluded multivariable adjustment for confounders; hence, the conclusion that treatment initiation after gestational week 12 is the optimal timing remains exploratory and needs validation in larger cohorts. Moreover, there was no parallel control group of patients who did not receive the “sandwich regimen”. Offspring follow‐up was only conducted up to 1 year postpartum, and extended surveillance is required to observe long‐term offspring immune function and developmental indicators. Future large‐sample multicenter controlled trials incorporating multiple laboratory biomarkers will optimize perigravid therapeutic regimens.

## 5 Conclusions

This multicenter prospective cohort study confirms that the three‐stage “sandwich” regimen combining preconception and postpartum B‐cell depletion with gestational bridging therapy of low‐dose azathioprine plus glucocorticoids achieves effective disease control and favorable maternal‐fetal outcomes in AQP4‐IgG‐positive patients with NMOSD exposed to B‐cell depleting agents before conception. No severe obstetric complications or adverse long‐term offspring developmental events were observed, preliminarily validating the clinical safety and feasibility of this full‐cycle perigravid management protocol.

## Funding

This study was supported by the Guangdong Medical Research Fund (B2026389), the Third Affiliated Hospital of Sun Yat‐Sen University, Clinical Research Program (QHYH202501), and the Cultivation Program for National Natural Science Foundation of China, the Third Affiliated Hospital of Sun Yat‐Sen University (2026GZRPYQN09). National Natural Science Foundation of China (82571540, 82071343, 82371354, 81701188).

## Ethics Statement

All participants (or their legal guardians for minors) provided written informed consent to participate in this study. The study protocol was approved by the Ethics Committee of the Third Affiliated Hospital of Sun Yat‐Sen University (approval number: [2021]02‐400‐01) and the medical ethics committee of the University of Hong Kong‐Shenzhen Hospital (approval number: [2024]200).

## Conflicts of Interest

The authors declare no conflicts of interest.

## Supporting information


**Table S1:** Maternal hematologic, hepatic, renal, and autoantibody laboratory parameters: Preconception, gestational and postpartum measurements.
**Table S2:** Peripheral blood cell counts and B‐cell proportions in offspring during follow‐up.
**Figure S1:** Infant growth trajectories (0–12 months) of 8 AQP4‐IgG‐positive NMOSD mothers' offspring rows 1–8 correspond to each mother's offspring by maternal identification number. Colored lines indicate WHO infant growth percentiles (P3, P10, P50, P90, P97). Black squares mark serial measured growth data at scheduled pediatric follow‐ups. Gray vertical bands represent routine pediatric developmental assessment intervals, with all infant growth metrics referenced to WHO standard 0–12 month child growth curves.
**Figure S2:** Pre‐conception and postpartum brain, optic nerve, and spinal cord MRI scans in 8 women with AQP4‐IgG‐positive neuromyelitis Each horizontal row corresponds to one patient by maternal identification number (Mat. ID 1–8); the left column group displays pre‐conception images, while the right column group shows postpartum images. For each participant, three imaging sequences are presented in fixed order: axial T2‐fluid‐attenuated inversion recovery (T2‐FLAIR) brain scans (transverse plane), coronal T2 fat‐saturated (T2FS) orbital sequences for bilateral optic nerves, and sagittal T2 fat saturated (T2FS) cervical‐thoracic spinal cord scans. No remarkable interval changes in demyelinating lesions within the brain, optic nerves, or spinal cord were identified between pre‐conception and postpartum time points in all subjects.

## Data Availability

Data will be made available on request.
