# Supplementary material for: Efficacy and Safety of Multidisciplinary Sandwich Regimen in AQP4‐IgG+ NMOSD With Preconception B‐Cell Depletion During Perigravidity
Source: CNS Neurosci Ther. 2026 Oct 23;32(10):e71222. doi: 10.1002/cns.71222 (PMC13692700; doi:10.1002/cns.71222)

Supplementary Table 1. Maternal Hematologic, Hepatic, Renal, and Autoantibody Laboratory Parameters: Preconception, Gestational and Postpartum Measurements[a]

Mat	1	2	3	4	5	6	7	8	Median
Preconception									
WBC (×109/L)	7.49	5.82	6.72	9.48	5.16	5.98	6.87	4.07	6.35
Hb (g/dL)	139	131	135	136	135	120	120	130	133.00
PLT (×109/L)	287	220	234	389	158	306	264	240	252.00
Absolute Lymphocyte Count (×109/L)	1.48	2.13	2.44	2.59	1.26	1.94	1.98	1.78	1.96
Absolute Neutrophil Count (×109/L)	4.76	3.06	3.56	5.86	3.41	3.51	4.07	1.66	3.54
ALT (U/L)	24	12	8	11	9	10	27	31	11.50
AST (U/L)	18	14	16	17	16	13	15	29	16.00
BUN (mmol/L)	2.78	2.87	2.16	4.10	3.61	5.15	4.98	4.83	3.85
CREAT (umol/L)	39	44.4	48.5	36.0	42.0	46.0	42.0	51.0	43.20
Serum AQP4-IgG titer[b]	1:320	1:32	1:320	1:100	1:100	1:32	1:320	1:100	-
Gestation									
WBC (×109/L)	9.45	11.37	8.56	9.42	8.93	8.54	9.11	8.68	9.02
Hb (g/dL)	126	116	131	142	125	128	129	133	128.5
PLT (×109/L)	281	209	163	320	147	320	285	334	283.00
Absolute Lymphocyte Count (×109/L)	1.2	1.74	1.18	1.13	0.79	1.82	1.89	1.69	1.44
Absolute Neutrophil Count (×109/L)	7.46	8.99	7.01	5.86	7.68	6.24	6.5	6.27	6.76
ALT (U/L)	11	12	10	12	9	21	12	22	12.00
AST (U/L)	15	22	14.8	12	15	14	15	16	15.00
BUN (mmol/L)	2.21	4.51	2.36	6.43	3.61	5.38	3.28	3.54	3.58
CREAT (umol/L)	36.0	43.5	46.5	38.0	42.0	53.8	46	26.7	42.75
Serum AQP4-IgG titer[b]	1:32	1:32	1:320	1:100	1:100	1:320	1:320	1:320	-
Postpartum									
WBC (×109/L)	6.99	6.79	7.37	6.45	8.11	6.39	8.94	11.68	7.18
Hb (g/dL)	113	117	120	144	134	124	129	125	124.50
PLT (×109/L)	363	147	101	381	162	310	297	267	282.00
Absolute Lymphocyte Count (×109/L)	1.38	1.46	1.31	2.4	1.76	1.83	2.20	1.27	1.61
Absolute Neutrophil Count (×109/L)	4.74	4.76	5.52	3.2	5.49	4.19	5.91	9.22	5.12
ALT (U/L)	33	15	7.8	19	9	15	27	13	15.00

AST (U/L)	22	24	11	19	16	20	19	13	19.00
BUN (mmol/L)	5.7	4.25	1.91	3.95	4.51	3.24	4.41	4.2	4.22
CREAT (umol/L)	50	42	50.7	40.0	47.0	46.0	33.0	39.6	42.75
Serum AQP4-IgG titer[b]	1:320	1:100	1:320	1:320	1:320	1:320	1:320	1:320	-

Abbreviations: WBC, white blood cell; Hb, hemoglobin; PLT, platelet; ALT, alanine aminotransferase; AST, aspartate aminotransferase; BUN, blood urea nitrogen; CREAT, creatinine; AQP4-IgG, aquaporin-4 immunoglobulin G.

[a]Blood samples were collected at three time points: preconception (within 3 months before conception), gestational (at 24 weeks of gestation), and postpartum (2 weeks after delivery).

[b]AQP4-IgG titer was determined by live cell-based assay and reported as endpoint dilution (1:x).

Supplementary Table 2. Peripheral Blood Cell Counts and B-Cell Proportions in Offspring During Follow-up

Child	1	2	3	4	5	6	7	8	Median
At Birth									
WBC（×109/L）	11.85	15.7	23.1	22.15	24.64	15.76	7.97	12.21	15.73
Hb（g/dL）	140	179	206	196	141	190	154	139	166.5
PLT（×109/L）	286	213	187	289	301	252	289	361	287.5
Absolute Lymphocyte Count（×109/L）	2.26	3.8	6.2	2.97	3.05	3.72	3.48	5.12	3.6
Absolute Neutrophil Count（×109/L）	7.83	9.6	15.2	17.36	19.62	10.38	3.02	5.28	9.99
B cells relative (%) (10.7% to 35%)	18.1	16.4	19.5	17.2	18.9	20.2	9.8	16.9	17.65
1 Month Old									
WBC（×109/L）	9.8	11.2	8.5	8.39	10.8	13.2	8.69	13.14	10.3
Hb（g/dL）	122	115	128	121	119	105	104	105	117
PLT（×109/L）	236	195	261	542	210	284	543	452	272.5
Absolute Lymphocyte Count（×109/L）	5.1	6.0	4.7	5.77	5.8	8.1	6.3	7.57	5.9
Absolute Neutrophil Count（×109/L）	2.4	2.9	1.8	1.58	2.6	3.8	1.08	3.32	2.5
B cells relative (%) (10.7% to 35%)	18.3	21.5	16.8	17.3	19.7	22.4	19.1	18.3	18.7

Abbreviations: WBC, white blood cell; Hb, hemoglobin; PLT, platelet.

Supplementary Figure 1. Infant Growth Trajectories (0–12 Months) of 8 AQP4-IgG-Positive NMOSD Mothers' Offspring

Rows 1–8 correspond to each mother's offspring by maternal identification number. Colored lines indicate WHO infant growth percentiles (P3, P10, P50, P90, P97). Black squares mark serial measured growth data at scheduled pediatric follow-ups. Gray vertical bands represent routine pediatric developmental assessment intervals, with all infant growth metrics referenced to WHO standard 0–12 month child growth curves.

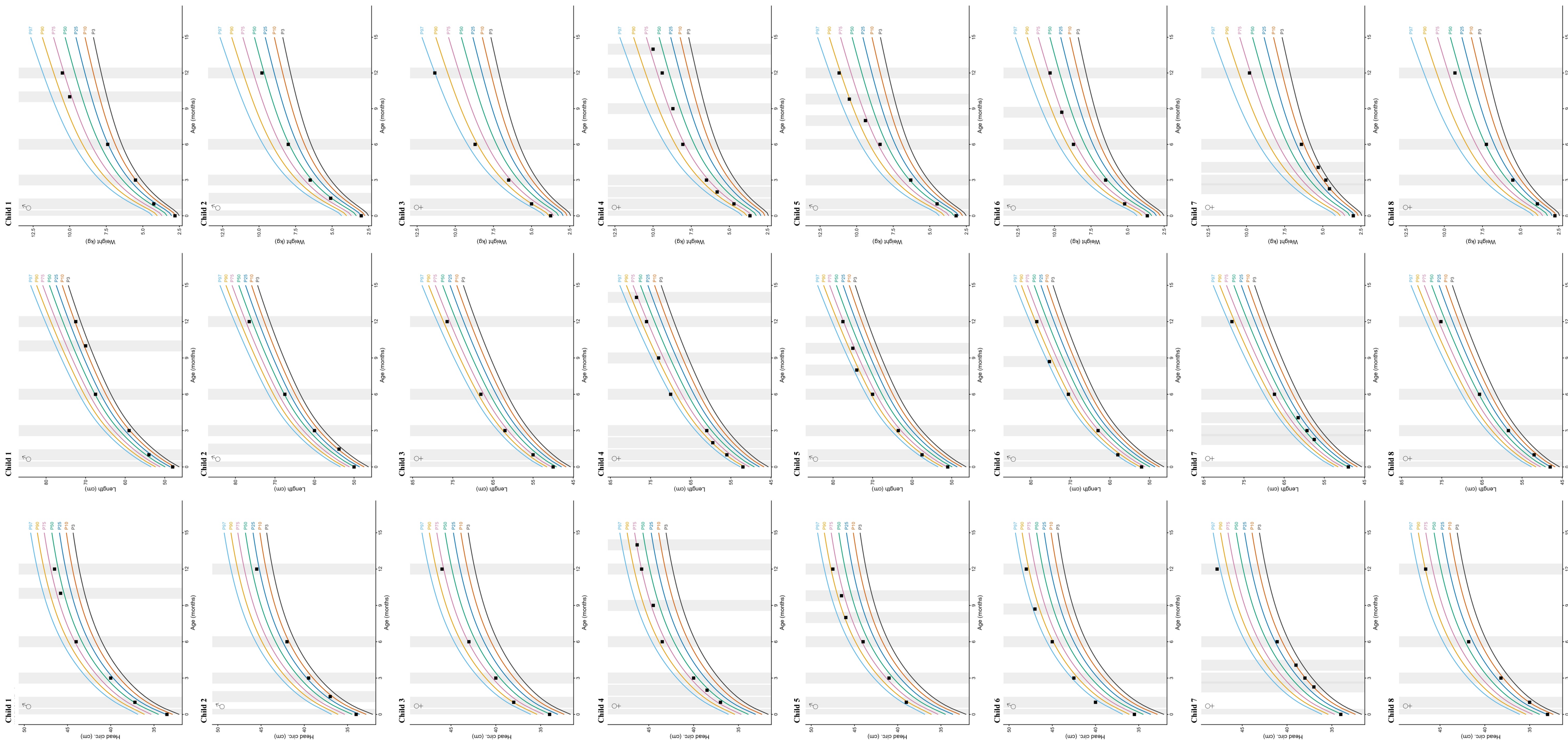

Supplementary Figure 2. Pre-conception and postpartum brain, optic nerve, and spinal cord MRI scans in 8 women with AQP4-IgG-positive neuromyelitis optica spectrum disorder (NMOSD)

Each horizontal row corresponds to one patient by maternal identification number (Mat. ID 1–8); the left column group displays pre-conception images, while the right column group shows postpartum images. For each participant, three imaging sequences are presented in fixed order: axial T2-fluid-attenuated inversion recovery (T2-FLAIR) brain scans (transverse plane), coronal T2 fat-saturated (T2FS) orbital sequences for bilateral optic nerves, and sagittal T2 fat-saturated (T2FS) cervical-thoracic spinal cord scans. No remarkable interval changes in demyelinating lesions within the brain, optic nerves, or spinal cord were identified between pre-conception and postpartum time points in all subjects.

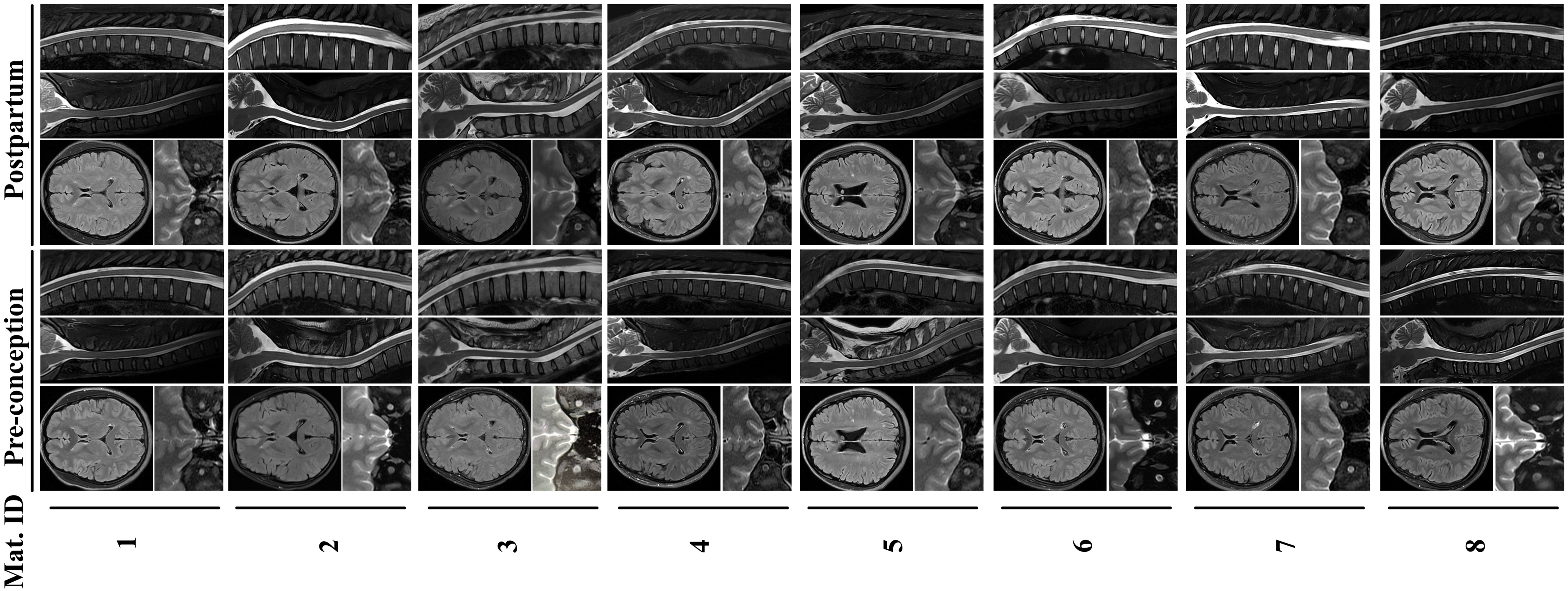

Supplement: Supplementary file 1 [file CNS-32-e71222-s001.pdf]
